# Mycobacterium chelonae infection following repeated mixed-agent cosmetic injections: A case report

**DOI:** 10.1016/j.jdcr.2026.04.022

**Published:** 2026-04-21

**Authors:** Xu Yuan, Shiliu Huang, Lin Gao

**Affiliations:** Department of Dermatology and Aesthetic Medicine, Honghui Hospital, Xi’an Jiaotong University, Xi’an, Shaanxi Province, China

**Keywords:** antimicrobial resistance, cosmetic injections, cutaneous and soft tissue infection, *Mycobacterium chelonae*, nontuberculous mycobacteria

## Introduction

*Mycobacterium chelonae* (*M chelonae*) is a rapidly growing nontuberculous mycobacterium (NTM) widely distributed in soil and water systems, exhibiting marked environmental resilience and opportunistic pathogenicity.^1^

With the rapid expansion of injectable cosmetic procedures, injection-associated *M chelonae* infections are increasingly recognized as a cause of postprocedural cutaneous and soft tissue infections,^2^ typically presenting as erythematous nodules, abscesses, or indurated plaques involving the skin and subcutaneous tissue, often accompanied by tenderness and regional lymphadenopathy.^3^ Owing to their indolent course and nonspecific manifestations, such infections are frequently misdiagnosed, requiring histopathologic examination, acid-fast staining, culture, and molecular testing for definitive identification.^3^

Because *M chelonae* exhibits intrinsic resistance to many conventional antibiotics, management often necessitates prolonged multidrug regimens, with most isolates remaining susceptible to macrolides, aminoglycosides, and selected fluoroquinolones despite emerging resistance trends.^4^ Long-term NTM treatment is frequently complicated by adverse drug reactions that may compromise adherence, increasing the risk of relapse or suboptimal response.^5^ In patients with baseline conditions predisposing to medication-related adverse effects, including gastrointestinal disorders or neurogenic tinnitus, these challenges may be amplified.^6^

## Case report

A 34-year-old woman presented with a 1-week history of multiple painful subcutaneous nodules on both lower extremities. Physical examination revealed approximately 11 erythematous, tender nodules distributed along previous injection sites, with localized warmth and bilateral inguinal lymphadenopathy.

**Fig 1:**
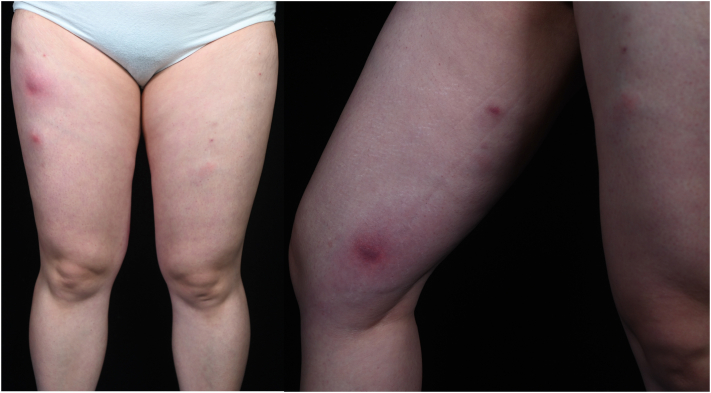
Multiple erythematous nodules and indurated subcutaneous masses distributed along prior injection sites on both lower extremities at presentation.

Three months earlier, she had received 3 sessions of “slimming injections” at a nonmedical facility. Mixed formulations containing at least 8 agents, including corticosteroids, epinephrine, sodium bicarbonate, and herbal components, were administered at multiple sites on both lower limbs. Although sterile draping was reportedly used during the first 2 sessions, standardized aseptic precautions were not implemented during the final session. One week after the last injection, progressive erythema and pain developed at the injection sites (Fig 1). Intravenous cephalosporins administered elsewhere provided minimal improvement.

Her medical history was notable for chronic neurogenic tinnitus and long-standing sleep disturbance and anxiety.

**Fig 2:**
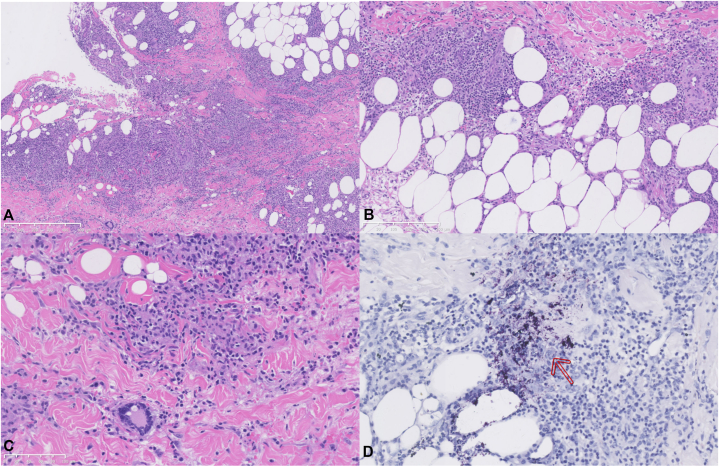
Hematoxylin–eosin staining demonstrates granulomatous inflammation involving the dermis and subcutaneous tissue. **A,** Granulomatous nodules. Scale bar = 500 μm. **B,** Involvement of the subcutaneous tissue with fat necrosis. Scale bar = 250 μm. **C,** Mixed inflammatory cell infiltration predominantly composed of histiocytes. Scale bar = 100 μm. **D,** Acid-fast staining demonstrates scattered acid-fast bacilli (*arrow*).

Laboratory evaluation showed mildly elevated C-reactive protein levels. Empirical therapy with minocycline (100 mg once daily) and clarithromycin (500 mg once daily) was initiated for suspected atypical mycobacterial infection. Skin biopsy demonstrated granulomatous inflammation involving the dermis and subcutaneous fat with fat necrosis and mixed inflammatory infiltrates (Fig 2, *A*-*C*); acid-fast staining was positive (Fig 2, D). Subsequent culture and polymerase chain reaction confirmed *M chelonae*.

**Fig 3:**
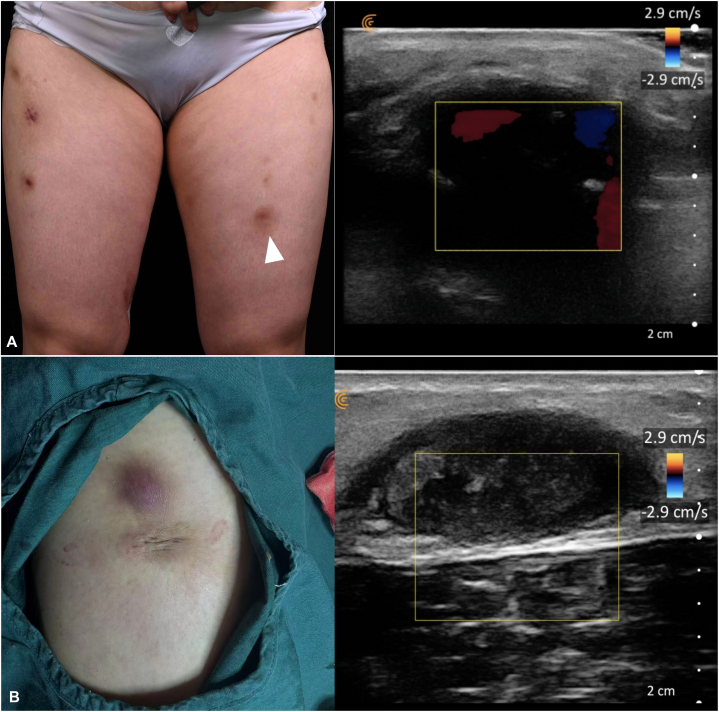
Doppler ultrasonography of the left thigh lesion. **A,** Prominent intralesional vascular signals after treatment interruption (*white triangle*). **B,** Disappearance of vascular signals after 4 months of continuous therapy.

Initial improvement was observed; however, therapy was discontinued for approximately 2 weeks because of worsening tinnitus and menstrual irregularities, followed by lesion flare and emergence of new nodules. After treatment was resumed, response was slower, with ultrasonography showing persistent inflammatory activity and increased vascular signals (Fig 3, A). Additional molecular testing was considered but declined for financial reasons.

Rifampin (0.45 g once daily) and ethambutol (0.25 g 3 times daily) were added, resulting in gradual clinical and imaging improvement over the subsequent 2 months (Fig 3, B). Image-guided surgical excision and debridement were then performed. After approximately 11 months of combined medical and surgical treatment, the lesions nearly resolved without recurrence. Mild tinnitus fluctuation persisted but did not preclude continuation of therapy.

## Discussion

NTM are environmental opportunistic pathogens increasingly implicated in cosmetic procedure–associated cutaneous infections.^7^ Rapidly growing mycobacteria, including *M chelonae*, present diagnostic and therapeutic challenges due to indolent course, antimicrobial resistance, and prolonged therapy.

In this case, repeated injections of mixed formulations in a nonmedical setting likely increased contamination risk, with the absence of standardized aseptic precautions temporally correlating with disease onset. Compared with single-procedure infections reported previously, repeated injections and mixed agents may further amplify infection risk.

Relapse following treatment interruption and attenuated response upon reinitiation underscore the importance of uninterrupted therapy in *M chelonae* infection. Potential contributors include emerging resistance, mixed infection, or host-related factors. In addition, limitations in culture and molecular sensitivity may hinder comprehensive pathogen detection, and identification of a single organism does not exclude coinfection.^8^

Standardized susceptibility testing for rapidly growing NTM remains unavailable in many clinical laboratories in mainland China, complicating individualized regimen optimization. Financial barriers may further restrict access to advanced molecular testing.

This case also highlights the impact of baseline comorbidities on treatment adherence. Neurogenic tinnitus contributed to temporary discontinuation, subsequent relapse, and the need for regimen intensification. Because prolonged multidrug therapy is often required, adverse effects and psychological burden may become significant barriers to sustained treatment, particularly in patients with pre-existing auditory or gastrointestinal conditions.^5^^,^^9^ For such patients, early risk assessment, close monitoring, multidisciplinary collaboration, and timely surgical intervention when appropriate may improve therapeutic continuity and disease control.

As injectable cosmetic procedures continue to expand, attention should extend beyond immediate complications to delayed infectious sequelae such as NTM infection. Persistent or atypical postinjection lesions unresponsive to conventional antibiotics should prompt early biopsy and molecular evaluation. During prolonged NTM therapy, adherence remains a critical determinant of outcome, with adverse drug reactions and underlying comorbidities potentially compromising treatment continuity and increasing the risk of relapse. Comprehensive, individualized management strategies are therefore essential to optimize outcomes.

## Conflicts of interest

None disclosed.
